# Radiological Parameters Review for Choanal Atresia

**DOI:** 10.3390/pediatric13020038

**Published:** 2021-06-01

**Authors:** Daniela Messineo, Maryia Chernikava, Valeria Pasquali, Serena Bertin, Mario Ciotti, Giulia de Soccio, Vincenzo Savastano, Carlo Catalano

**Affiliations:** 1 Radiology, Oncology, and Anatomopathological Department, University La Sapienza of Rome, 00185 Rome, Italy; chernikava.1797210@studenti.uniroma1.it (M.C.); pasquali.1061237@studenti.uniroma1.it (V.P.); mario.ciotti@uniroma1.it (M.C.); carlo.catalano@uniroma1.it (C.C.); 2 UOD Pediatric ENT ‘Umberto I’ Policlinic, 00185 Rome, Italy; s.bertin@policlinicoumberto1.it (S.B.); giulia.desoccio@gmail.com (G.d.S.); v.savastano@policlinicoumberto1.it (V.S.)

**Keywords:** computer tomography, choanal atresia, measure, surgery

## Abstract

(1) Background: The study aims to identify which imaging parameters are necessary for a new correct surgical approach in the study of choanal atresia, and which anatomical findings are essential for correct planning of endoscopic treatment in choanal atresia. (2) Methods: In this retrospective study, 19 patients with choanal atresia had high-resolution multiplanar imaging (14 cases aged ≤1 year and 5 cases aged 1 to 3 years) and 35 patients in the control group similarly distributed by age. Fourteen variables, the most relevant from a surgical point of view, were selected and measured. A comparison was made between the averages of the study group and the different control groups, either directly observed or selected from the literature, using Pearson’s correlation. (3) Results: In 14 out of 26 cases, the differences were statistically significant. There was a correlation between the structures assessed, such as choanal height, rostrum height, and age. (4) Conclusions: Thanks to volumetric reformatting, this work identified and provided the clinician with useful information that helped choose the correct surgical approach. Furthermore, it focused on which imaging parameters are necessary to improve the planning of the surgical correction of choanal atresia.

## 1. Introduction

Choanal atresia is a rare congenital disorder caused by a failure to develop the posterior nasal cavity (choana), resulting in a missing opening between the nasopharynx and the nasal cavities. The incidence of this malformation reported in the literature is approximately 1/5000–7000 live births [1].

Generally, choanal atresia may affect one or both Choanae, although most studies show that the unilateral form is more common than the bilateral one [2,3], and the incidence is higher in females than in males (2:1) [4,5]. The unilateral form is frequently on the right side [2,5]. Bilateral atresia is more commonly associated with syndromes such as CHARGE, Treacher-Collins, and Crouzon’s disease [4,6]. Bilateral atresia is a congenital disease that represents a surgical emergency. It is severe because the infant, during the first month of life, breathes preferentially through the nose. For this reason, this disorder usually presents with severe symptoms and can cause respiratory failure with intercostal and retrosternal retraction, abnormal movement of the wing nose during breathing, dyspnea, cyclic cyanosis, and asphyxia. On the other hand, children subject to unilateral breathing will have late unilateral nasal obstruction, persistent ipsilateral rhinorrhea, and recurrent rhinosinusitis.

Parietal atresia consists of a blockage resulting from a thickening of the parietal bone: the vomer medially, the pterygoid plate laterally, which may be fused or divided by a fi-bro-periosteal layer covered by mucosa. Previous studies have shown that the ratio of bone to membranous atresia is 9:1; although a detailed review of CT findings with histopathological studies showed that mixed-wall atresia is the most common and is present in 71% of cases, while in 29% of cases we found a pure bone wall [7,8].

The treatment of end-tidal atresia is exclusively surgical. The most commonly used surgical procedures consist of a transnasal endoscopic approach. The ENT practitioner prepares the posterior part of the nasal septum, the cartilaginous bony part of the rostrum, and the mucosa and performs resection of the atresia wall.

To define the prominent anatomical landmarks to plan a correct endoscopic approach for atresia, we chose a set of parameters of the whole cavity and compared them with negative controls with the same age distribution. In addition to the clinical parameters, we identified the rostrum height, width, and height. All parameters mentioned above represent essential knowledge for planning the surgical procedure.

## 2. Materials and Methods

This study was conducted by sophisticated head and neck radiology operating unit and pediatric department of a tertiary referral center. In the study, we included 19 patients with choanal atresia. The mean age of the children was 207 ± 333 days; 14 were less than 1 year old and 5 between 1 and 3 years; 10 were females and 9 males; 5 were affected by the unilateral form on the right side and 10 by the bilateral form. These cases were reported for more than 10 years (2008–2019). The control group consisted of 35 children without craniofacial malformation, with a mean age of 507 ± 418 days, 16 of whom were less than 1 year old, and the others were between 1 and 3 years old. These details are summarized below (Table 1). In this type of study, children with genetic malformations were excluded.

Our study began with a review of the literature and identified the points necessary for the diagnosis to be reported. To this end, we selected two sets of data: the first by examining children who had the same characteristics as those affected by atresia (age and no genetic malformations), the second, from three authors whose reference points were helpful for reporting—see Table 2 (Aslan, 2008 [9]; 3: Likus, 2014 [10]); 

These young patients underwent a high-resolution volumetric HRCT scan (CT Aquilion, Toshiba, channels). The standard parameters used for the test depended on the patient and ranged between 120 and 100 kV, in 100–50 mA with a step of 1–0.5 s per rotation, followed by a multiplanar reconstruction (MPR) of approximately 8 mGy.

To avoid movement artifacts or lack of compliance on young patients, we opted for a whole stomach window after the baby’s feeding, solving the problems with MPR.

We meet the technical correctness criteria for HRCT imaging to standardize data acquisition, which, for axial scanning, are the symmetry of the visible structures, the reconstructive plane must make parallel to the plane of the hard palate, as well as for sagittal and coronal reconstructions, care must also be taken to a position perpendicular to it.

**Figure 1:**
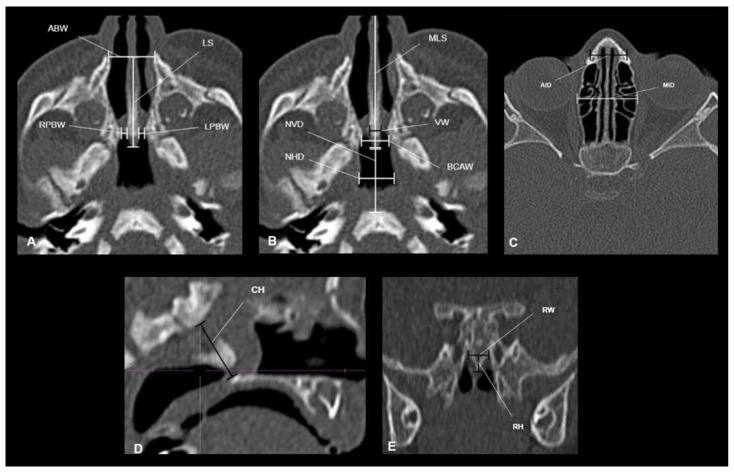
(**A**–**C**) Summary of measurements in the axial plane (LS: Septum length; ABW: Anterior bone width; RPBW: Right posterior bone width; LPBW: Left posterior bone width; MLS: Maximum septum length; VW: Septum thickness; BCAW: Choana bone width; NVD: Vertical distance of the nasopharynx; NHD: Horizontal distance of the nasopharynx). B. Summary of anterior axial plane measurements (AID: Anterior interorbital distance; MID: Mean interorbital distance). (**D**). Measurement of choanal height (CH) in the sagittal plane. (**E**). Measurement of the rostrum in the coronal plane (RW: rostrum width; RH: rostrum height).

We measured 14 different dimensions of the nasal and nasopharyngeal cavities (Table 3). Eleven variables were identified based on pre-existing scientific findings, which we considered clinically valuable [9,10,11,12] (Figure 1A–C). Additionally, we measured the height, width, and height of the rostrum (Figure 1D,E).

All parent patients enrolled gave their consent to the study and use of data for research.

We calculated the Pearson correlation. As the measurements are considered to be closely related to the age of the children, we decided to compare our study group with the control group by stratifying the data into two groups:

− Children with an age range <1 year;
− Children with an age range of 1–3 years.

For each of these 14 measures, the mean and standard deviation were calculated, and then the mean of the study group was compared to the negative controls in the scientific literature utilizing the Student’s *t*-test. Specifically, channel height, rostrum width, and rostrum height were analyzed with our control group, while the comparison of the remaining 11 variables was carried out using data from the literature [5,6,10]. Differences with probability <0.05 were considered statistically significant.

All enrolled parent patients gave their consent to the study and the use of the data for the research.

## 3. Results

A direct relationship between the variables (CH, RW, RH) was noted by analyzing the Pearson correlation coefficient. As age increases, there is an increase in the measures considered, to be noted in the table with the positive sign. From this consideration, it can be concluded that there is a strong correlation between choanal height and age in both groups (R^2^ = 0.77 and R^2^ = 0.69, respectively) and that there is a poor correlation with rostrum width, especially in the study group (R^2^ = 0.31 and R^2^ = 0.37), and a good correlation with rostrum height (R^2^ = 0.60 and R^2^ = 0.50) (Table 4).

For each of the 14 variables, a comparison was made between the averages of the study group and different control groups, either directly observed or selected from the literature (Table 2). Specifically, channel height, rostrum width, and rostrum height were analyzed with data from their control group, while normative data available in the literature were taken for the comparison of the remaining 11 variables [9,10,12]. The REF column indicates which control group was considered, which can be found at the bottom of the Table 2. As already mentioned, the tests were carried out by distinguishing two age groups.

**Figure 2:**
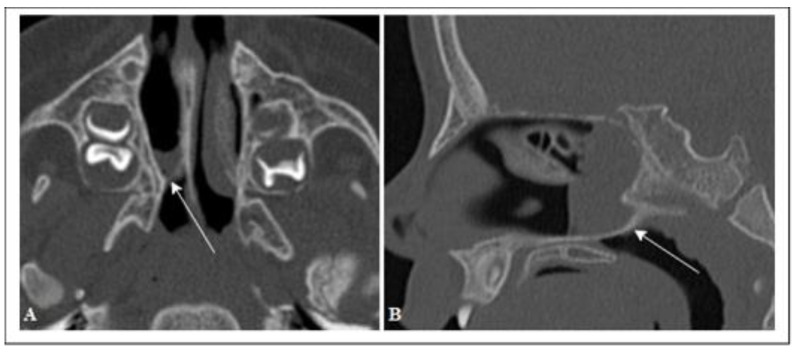
Axial CT scan (**A**), sagittal reconstruction (**B**) of a child with right unilateral choanal atresia. Mixed atresia wall; bone (arrows).

In 16 of the 26 comparisons, the differences were statistically significant (Table 4). The DX septum type was mucous in 68% (13 patients) of the cases, bony in 3 cases, and mixed in 3 others. The SN septum type was not detectable in 5 cases (26%); in the remaining cases, it was mucous (42%), bony (16%), and mixed (16%) (Figure 2).

**Figure 3:**
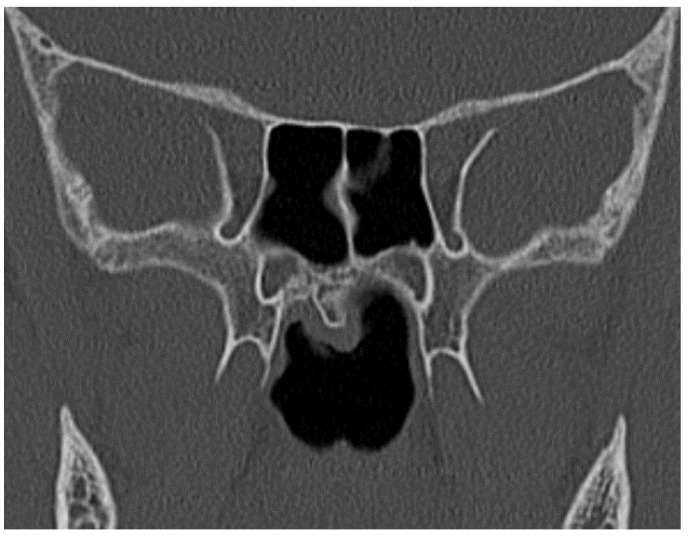
Eccentric and dysmorphic rostrum.

Only one case of rostrum dysmorphia was found that was eccentric and had increased in size (Figure 3).

## 4. Discussion

Choanal atresia is a rare congenital malformation resulting from the failure of the posterior nasal cavity (cohane) to develop, with consequent failure of the nasal fossae to communicate with the nasopharynx. It can be responsible for respiratory distress in infants, especially in bilateral forms. Its management represents a surgical emergency. Multilayer computed tomography is an excellent tool for the characterization of this pathology and highlighting other congenital anomalies that may be associated. Atresia obstruction generally affects the posterior part of one or both nasal cavities. It is due to the medicalization of the medial lamina of the pterygoid apophysis and lateralization and thickening of the posterior portion of the vomer, which may be fused together or separated by a mucosa-covered fibro-periosteal layer [1,13].

The choanal atresia can cause respiratory distress in the newborn, particularly the bilateral form. Management of the patient is a surgical emergency. Multislice CT is an excellent tool to evaluate this disorder and to detect other associated congenital anomalies. Atresia obstruction usually affects the posterior aspect of one or both nasal cavities. It is due to the medicalization of the medial pterygoid plate and the lateralization and thickening of the posterior part of the vomer, which can lead to fusion of these two parts, but they can also be separated by a fibro-periosteal layer covered by membranous tissue [1,13].

In order to obtain a more precise anatomical definition of choanal atresia, in 1985, Slovis et al. analyzed the CT scans of 11 patients with choanal atresia and 66 negative control patients and established two valuable parameters for the assessment of this disease (i.e., the width of the vomer and the distance of the airway from the Choanae). According to their study, the average width of the vomer in negative patients from 0 to 8 years was 0.23 cm, and at the age of 20 years, it reached 0.28 cm. The distance between the airway and the Choanae, which was described as the distance between the lateral walls of the nasal cavity and the vomer, was about 0.67 cm and increased by 0.027 cm/year for the first 20 years. In the case of bone atresia, the choanal airway was absent, while in the membranous form, it was reduced by about 1/3 of the standard size. The width of the vomer in bone atresia is about 0.6 cm, and in the case of membranous, it is 0.3 cm [14]. It is essential to emphasize that the age variety of the Slo-vis group implies that their study cannot overlap with ours since, during the growth years, the fluctuation of our variable is too significant. However, in the past decades, this study laid the foundations for surgical procedures, and it is a good reference point for surgeons.

Today, the introduction of new techniques, such as, but not limited to, endoscopic procedures and flexible tubes, requires new anatomical landmarks.

Waitzman et al., in their study, evaluated whether CT measurements of the upper craniofacial skeleton accurately represent the bone region. For this, they compared direct measurements of bone structures taken on dry skulls with axial CT images of the same. Excellent agreement was found between the direct (dry skull) and indirect (CT) measurements. Furthermore, they showed that the error was within clinically acceptable limits (less than 5 percent) if the angle was no more than ±4° from baseline (0°). The authors emphasized that craniofacial measurements obtained from CT scans are accurate, reproducible, and can be used to aid diagnosis and planning of surgery [12]. In another retrospective study conducted by the same authors, 15 craniofacial skeletal measurements were taken from 542 sets of CT images of normal subjects. The values of the measurements were then divided into age categories of 1 year from 1 to 17 years and into four age groups for those younger than 1 year. The authors presented the normal range and growth patterns of measurement values for the cranial vault, orbital region, and upper midface. The authors conclude that knowledge of growth patterns and typical measurement values in the craniofacial region will help to improve diagnostic accuracy and pre-surgical planning [11].

They concluded that knowledge of the craniofacial region would improve diagnostic accuracy and preoperative planning [11]. This study represented the first comparison for categories, including children aged 1–3 years for assessment of anterior and median inter-orbital distance. The homogeneity of the category allowed us to have comparable measures; it should be noted that the measures obtained today have a similar role; in fact, the surgical approach requires more information about the internal anatomy of the nasal cavity rather than the outer part.

Cheung et al. studied the craniofacial bone characteristics of 8 children with bilateral choanal atresia and compared them with 13 negative controls who were less than 3 months old, taken from the Waitzman study [11]. They compared 14 cranio-orbital zygomatic features to portray the configuration of the craniofacial bones. They showed that 10 of the 14 features were statistically significant between the study group and the control group. In their conclusions, they point out that infants with bilateral choanal atresia may have a proportionally smaller cranio-orbital zygomatic skeleton than the healthy population [11,15].

Different dimensions of the structure of the nasal cavities were established in the research conducted by Aslan et al., comparing 9 children with bilateral choanal atresia and 104 children aged less than 1 year without nasal patency problems. The authors also showed that the width of the vomer and its surface area, like the width of the posterior apertures, were significantly different [9]. Although the measurements were made only in the axial plane, in our study, we tried to make measurements in the three fundamental planes, thanks to technological development and a multiplanar and three-dimensional approach of modern imaging. Likes et al. carried out a complex morphometric assessment of the bone and mucosal structures of the nasal cavities in relation to the age and sex dependency of children up to 3 years of age. A total of 180 children, aged between 0 and 3 years, were divided into 5 groups, and 18 distances between the bony and mucous structures of the nasal cavities were measured. The authors determined that there were no significant differences between the morphometric parameters analyzed in the adjacent age groups. The differences were only statistically significant in the extreme age groups.

Furthermore, they demonstrated a correlation between the size of the structures and age. Their results are a valuable supplement of morphometric data for the nasal cavities in young children [10]. This study shows no correlation with the most frequent pathological pictures of the nasal cavity.

Despite a thorough examination of the axial dimensions of the nasal cavity in the studies mentioned above, Fitzpatrick et al. focused on the parameters of the skull base as an area of high intraoperative risk since, at that age, most of the skull base is still made of cartilage and ossification is not completed until first years of age. They examined 13 patients with bilateral choanal atresia, three of whom were associated with CHARGE syndrome and 10 of whom had the isolated form. Choanal width, choanal height, median-nasal skull base height, and skull base steepness were assessed. No significant differences were found between the groups as the cohort was too small [16].

Our study differs from Fitzpatrick’s study in that we evaluated a more detailed set of nasal cavity measurements. Furthermore, we compared the measurements (choanal height) with control groups, bearing in mind the age-dependency of these variables. The aim of our study was to obtain data that could be relevant from a surgical point of view. This is the reasoning that led us to select 14 variables. In 16 of the 26 comparisons, there were statistically significant differences between the group studied and the control group. In agreement with previous studies, we confirmed the thickening of the vomer, the horizontal and vertical narrowing of the nasopharynx, and the smaller choana bone width in the age group up to 1 year. Anterior bone width, anterior and median interorbital distance, and maximum septal length were smaller in patients with choanal atresia in both age groups, just as Cheung and Aslan stated. The most widely used surgical procedure today requires a trans-nasal endoscopic approach. It requires resection not only of the septum part but also excision of the posterior third of the nasal septum, with resection of part of the osseocartilaginous rostrum and provision of mucosal flaps. In order to define the anatomical landmarks essential for proper planning of the endoscopic procedure for cohanal atresia, we included measurement of rostrum height, which were significantly different between the two groups analyzed. We also found a positive correlation with the age of the child.

Soft tissue measurements were not performed for CT assessment of the district, as they were considered clinically irrelevant due to the vital fluctuation of soft tissue thickness, which can be influenced by various factors. Furthermore, it is essential to emphasize the purpose of nasal decongestant for the differential diagnosis of mechanical nasal obstructions, such as choanal atresia, adenoid hypertrophy, septal deviation from inflammatory ones such as turbinate hypertrophy.

We did not measure the atretic plaque because it is the thinnest part and cannot be measured accurately. In addition, in some patients, the characterization of the causes and thickness of the atretic wall may not be accurate. Soft tissue adjacent to the bony septum may be from the density, even if the nasal cavities washed by nasal secretion and administered a vasoconstrictor argument.

**Figure 4:**
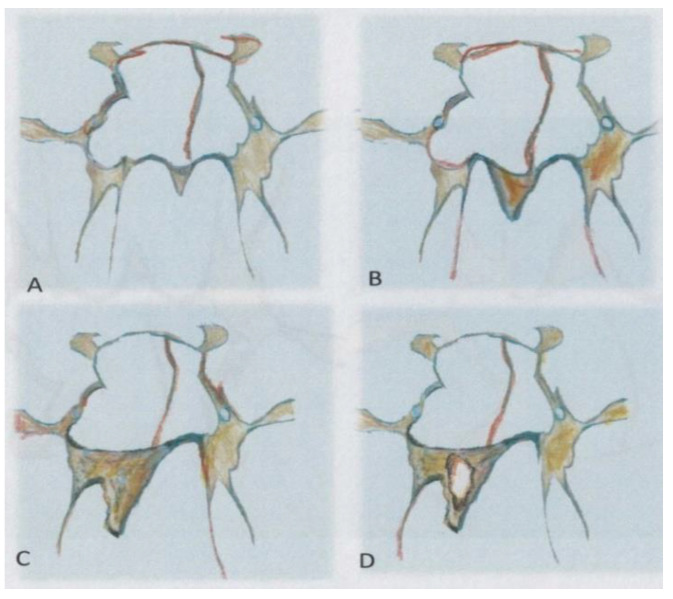
(**A**) Symmetric rostrum; (**B**) Asymmetric rostrum, (**C**) Eccentric, and Dysmorphic rostrum, (**D**) Pneumatized rostrum.

If we compare our work with previous studies, we can see that, previously, it was not necessary to know the particular anatomical landmark used by endoscopists or the height of the rostrum. In our experience, a classification of the rostrum was assumed (Figure 4), i.e., a rostrum with regular choana width that was asymmetrical and dysmorphic. Asymmetrical: when the widths of the choanal fossae are the same on the right and left, asymmetric when they are not, and the rostrum can be increased in size asymmetrically (dysmorphic), as was identified in one of our patients. We also theorized that a form of dysmorphic rostrum could also be found as in other bones of the sinus complex, but we had no cases of this.

## 5. Conclusions

This work has established that radiological imaging must search for anatomical parameters in order to plan and surgically correct choanal atresia.

In-depth anatomical knowledge is required for the use of new endoscopic techniques. This knowledge has allowed us to enhance our role, especially in preoperative planning, and to propose three rostrum morphologies that, in our opinion, should be reported for correct endoscopic planning.

## Figures and Tables

**Table 1:** Observed Population.

	Age Ranges	Average Age (Days)	Females	Males	Total
**Study group**	≤1 year	46.79 ± 98.39	8	6	14
	1–3 year	657.20 ± 354.09	2	3	5
	Total	207.42 ± 333.34	10	9	19
**Control group**	≤1 year	151.94 ± 82.20	8	8	16
	1–3 years	806.05 ± 344.35	6	13	19
	Total	507.03 ± 418.40	14	21	35

**Table 2:** Comparison of averages of patients with choanal atresia and control groups.

Measurements	Age	Study Group			Control Group			*p*-Value	REF.
		N	Media (mm)	Std. Dev.	N	Media (mm)	Std. Dev.		
Choanal height (CH)	≤1 year	14	8.1	1.6	16	12.0	1.9	<0.001	[1]
	1–3 years	5	14.2	1.3	19	16.2	2.5	0.103	[1]
Rostrum width (RW)	≤di 1 anno	14	6.3	1.2	16	6.3	1.1	0.932	[1]
	1–3 years	5	8.2	2.8	19	7.9	0.9	0.731	[1]
Rostrum height (RH)	≤1 year	14	3.6	0.7	16	5.1	1.0	<0.001	[1]
	1–3 years	5	7.0	2.3	19	6.6	1.3	0.640	[1]
Anterior interorbital distance (AID)	≤1 year	14	11.3	2.1	104	17.8	1.4	<0.001	[2]
	1–3 years	5	14.9	3.7	65	18.3	1.8	<0.001	[4]
Inter-orbital distance average (MID)	≤1 year	14	14.8	2.2	104	16.8	1.5	<0.001	[2]
	1–3 years	5	20.6	2.1	65	19.2	1.9	0.118	[4]
Bone septum thickness (VW)	≤1 year	14	4.9	1.2	104	2.2	0.6	<0.001	[2]
	1–3 years	5	3.5	0.9	69	3.7	0.8	0.665	[3]
Septum length (LS)	≤1 year	14	28.4	2.2	104	27.6	4.5	0.500	[2]
	1–3 years	5	38.4	3.9	69	38.2	2.9	0.923	[3]
Maximum septum length (MLS)	≤1 year	14	40.1	2.8	111	45.4	5.4	0.001	[3]
	1–3 years	5	47.5	11.4	69	53.2	4.5	0.018	[3]
Anterior bone width (ABW)	≤1 year	14	13.9	1.9	104	14.3	1.6	0.343	[2]
	1–3 years	5	17.2	3.8	69	18.6	1.3	0.055	[3]
Posterior right bone width (RPBW)	≤1 year	14	2.1	1.4	111	7.3	0.7	<0.001	[3]
	1–3 years	5	4.6	1.8	69	8.6	0.9	<0.001	[3]
Posterior left bone width (LPBW)	≤1 year	14	2.3	1.4	111	7.2	0.9	<0.001	[3]
	1–3 years	5	8.0	2.7	69	8.5	0.6	0.193	[3]
Bone width of choana (BCAW)	≤1 year	14	9.4	2.3	104	13.2	1.4	<0.001	[2]
	1–3 years	5	16.6	1.5	69	19.8	1.5	<0.001	[3]
Vertical distance nasopharynx (NVD)	≤1 year	14	16.9	4.0	104	20.7	3.2	<0.001	[2]
	1–3 years	5	25.8	5.3	nd				
Horizontal nasopharynx distance (NHD)	≤1 year	13	10.5	1.7	104	11.6	2.0	0.046	[2]
	1–3 years	5	16.2	1.3	nd				

References: 1: Own control group; 2: Aslan, 2008 [9]; 3: Likus, 2014 [10]; 4: Waitzman, 1992 [11].

**Table 3:** Parameters observed.

Measurements	Description
Choanal height (CH)	The distance between the horizontal lamina of the palatine bone and the body of the sphenoid
Rostrum width (RW)	The maximum width of the sphenoid triangular bone spine.
Rostrum height (RH)	The distance between the body of the sphenoid and the junction point of the vomer wings
Anterior interorbital distance (AID)	The distance between a point on each tear bone that represents the anterior end of the medial orbital wall
Mid Interorbital Distance (MID)	The distance between a point on each medial wall of the bone orbit (ethmoid bone) halfway between the torn bone and the base of the optical pillar
Septum thickness (VW)	The maximum width of the vomer
Septum length (LS)	The distance from the pyriform opening to the rear end of the vomer
Maximum septum length (MLS)	The maximum septum length from the most anterior part of the nasal septum to the rear end of the vomer
Anterior bone width (ABW)	The distance between the two ridges protruding from the anterior wall of the maxillary bones
Bone width of choana (BCAW)	The distance between the two pterygoid processes
Posterior right bone width (RPBW)	The distance between the lateral bone wall of the right nasal cavity and the mucosa of the septum
Posterior left bone width (LPBW)	The distance between the lateral bone wall of the left nasal cavity and the mucosa of the septum
The vertical distance of the nasopharynx (NVD)	The distance between the rear vomer and the base of the skull
Horizontal nasopharynx distance (NHD)	The distance between the middle third of the nasopharyngeal sidewalls

**Table 4:** Pearson’s correlation coefficient.

Coeff. Correlation R^2^	Correlation with Age (in Days)	
	Study Group	Control Group
Choanal height (CH)	0.77	0.69
Rostrum width (RW)	0.31	0.47
Rostrum height (RH)	0.60	0.50

## Data Availability

The scientific guarantor of this publication is Daniela Messineo. The dataset will be made available by making a request to Messineo, Daniela.messineo@uniroma1.it according to Italian law—and having reported the CT setting has a copy with authorization from the parent of the young patients.

## Abbreviations

CHARGE	coloboma of the eye: heart defects: atresia of the nasal choanae, retardation of growth and development, genital and urinary abnormalities, and ear abnormalities, and deafness syndrome
CT	computer tomography
HRCT	High-resolution computer tomography
MPR	multiplanar reconstruction
CH	Choanal height
RW	Rostrum width
RH	Rostrum height
REF	reference
